# Patient-specific instrumentation combined with a new tool for gap balancing is useful in total knee replacement: a 3-year follow-up of a retrospective study

**DOI:** 10.1186/s13018-021-02467-6

**Published:** 2021-05-12

**Authors:** Ting Deng, Tangyou Liu, Qing Lei, Lihong Cai, Song Chen

**Affiliations:** 1 Department of Orthopaedics, The Third Hospital of Changsha, Changsha, China; 2 Department of Radiology, The Third Hospital of Changsha, Changsha, China

**Keywords:** Total knee arthroplasty, Gap balance, Ligament balance, Balancer device, Flexion-extension gap surgical technique, Equipment design, Implantation technique, Measured resection technique

## Abstract

**Objective:**

The purpose of this study was to determine whether the gap-balancing technique with patient-specific instrumentation (PSI) and a new balancing device in total knee arthroplasty (TKA) can improve knee function to a greater extent than can the measured resection technique.

**Materials and methods:**

Data from 150 patients who underwent TKA from August 2014 to June 2016 were studied retrospectively. The gap-balancing technique assisted by PSI and the new balancing device was used in 80 patients (82 knees), and the measured resection technique was used in 70 patients (70 knees). The surgical, imaging, and knee function data were compared.

**Results:**

The gap-balancing technique assisted by PSI and the new balancing device was found to be feasible in all operated knees and reliable. In total, 150 patients (152 knees) of ages ranging from 52 to 78 years (mean 67 years) underwent TKA during the study period. The follow-up period ranged from 35 to 52 months (mean 45 months). Only one patient, who was included in the gap-balancing group, underwent a revision surgery at 2 years postoperatively due to infection. There were no differences in the incidence of anterior knee pain between the two groups. The mean flexion angle, KSS scores, and VAS scores did not significantly differ between the measured resection group and gap-balancing group at 12 weeks or 36 weeks postoperatively. The average joint line displacement was 1.3 ± 1.1 mm (range 0–3) proximally in the GB (gap-balancing) group and 1.2 ± 1.4 mm in the MR (measured-resection) group. No outliers >5 mm in either group were recorded. The mean leg axis deviation from the neutral mechanical axis was 1.8°±1.5° varus (range 0°–3°varus) versus the neutral mechanical axis in the GB group and 1.4°±1.2°(range 0°–3°)in the MR group. No outliers with >3° deviation in either group were recorded.

**Conclusions:**

The gap-balancing technique performed with the new balancing device and PSI can yield accurate femoral component alignment as well as outcomes similar to those of measured resection at 3 years. The new balancing device can be taken into consideration by surgeons who prefer performing the gap-balancing technique with PSI.

## Introduction

Total knee arthroplasty (TKA) is considered the most successful surgical treatment for end-stage knee osteoarthritis available in the twenty-first century. A successful knee replacement hinges on appropriate soft-tissue balancing and accurate bony alignment. With ideal limb alignment and soft-tissue balance, patients may be able to regain near-normal knee function, avoiding the early TKA failure caused by uneven forces being exerted on prosthesis and cement, and forces under the maximum limit ensure the integrity of the extensor mechanism [1, 2]. Previous discussions on the TKA surgical technique have focused on how to assess femoral component rotation. Femoral component rotational malalignment may lead to patellofemoral complications, such as abnormal patellar tracking, knee anterior pain, and joint adhesion, and these complications can worsen the levels of instability, function, and wear [3–5].

The rotational alignment of the femoral component involves the bony anatomy and soft tissue [3, 6]. There are two standard surgical techniques for prosthesis implantation that are utilized in TKA: measured resection and gap balancing.

The measured resection technique preserves the joint line postoperatively, has a short learning curve, and is a simple operation. Most surgeons use bony landmarks such as the transepicondylar axis, anteroposterior axis, or posterior condylar axis to determine the angle of femoral component rotation when using the measured resection technique [7, 8]. Some researchers believe that the measured resection technique is inaccurate due to inter-individual variations in femoral anatomy; it is difficult to determine the angle of femoral component rotation by TEA (transepicondylar axis), the AP axis (anteroposterior axis), or the PC axis (posterior condylar axis) [9]. It is often difficult to accurately locate the medial and lateral epicondylar bony landmarks intraoperatively [10–12]. The measured resection technique can lead to implant instability, as the technique has been suggested to have a higher incidence of femoral condylar lift-off than the gap-balancing technique [7, 13]. Dennis analyzed the bony landmark data that were recovered before resectioning by computer navigation of 212 TKA patients. The results showed a higher variability in the femoral component position using the TEA and only 43% of cases had a balanced alignment within ±3, of which the PC axis was 58% and AP axis was 39 %[14].

Freeman et al. first proposed the gap balancing technology in 1970 with the flexion gap [15], and Insall et al. improved this technique and proposed gap balancing technology that involved balancing extension first [16]. The gap-balancing technique relies on ligament release prior to bone cutting. The limb can obtain correct approximate alignment before femoral component rotation is performed by soft tissue release. Some studies have suggested that gap balance can lead to higher short-term satisfaction among patients [14, 17, 18]. Appropriate soft tissue release and accurate osteotomy of the tibia are essential when surgeons perform the gap-balancing technique because tibial resection serves as a basis and reference for femoral bone resections, especially in the extension gap-first technique. Inaccurate proximal tibial resection leads to a raised joint line, increased internal rotation, or excessive external rotation of the femoral component or a mismatch between the flexion and extension gaps [19].

With the development of 3D printing techniques and digital techniques, patient-specific instrumentation (PSI) has been widely applied in orthopedic clinics [20], and personalized 3D-printed resection blocks can be produced preoperatively based on MRI and CT data. However, the accuracy of PSI is still controversial. It has been proved by some studies that digital techniques are convenient and can produce PSI components that are precise and secure enough to be used in complex and delicate surgeries [21–23]. While other reports have showed that PSI deviated from the positions planed before the surgical plan by 10.5% in the coronal plane and 29.9% in the sagittal plane [24]. One of the causes for controversy is that most PSI systems used at present are only bone-referenced and barely consider a combination of functional parameters [25, 26]. The degree of soft tissue release that is appropriate is subjective and difficult to determine in the surgeries that adopt the gap-balancing technique.

To overcome the limitations of the gap-balancing techniques, we used PSI combined with a new gap-balancing device in TKA and compared this technique with the measured resection technique. There were three purposes of this study. The first purpose was to introduce a new, combination method for gap balancing in TKA. The second was to introduce a new tool to perform flexion gap balancing. The third was to evaluate implant survivorship, patient outcomes, complications, and radiographic parameter in patients who underwent TKA of these two groups.

## Patients and methods

### Ethical approval

Before we used the PSI and the new balancing device, we obtained approval from the Changsha No. 3 Hospital ethics committee and 3D printing technology medical application research institute of Changsha. Written informed consent was obtained from each patient in the gap-balancing group after the details of this study were explained orally.

### Study design

Data on the TKA surgeries performed at the Changsha No. 3 Hospital were retrieved. A total of 150 cases (152 knees) who underwent cemented primary TKA between August 2014 and June 2016. Most TKAs that were performed during this period were performed with one of two surgical techniques: the traditional measured resection technique or the gap-balancing technique assisted by a new balancing device and PSI. We collected the clinical data of these patients for retrospective analysis. **The study enrolled** patients aged 22–85 years with noninflammatory degenerative joint disease who were suitable candidates for cemented primary TKA. **The exclusion criteria** were as follows: patients who had inflammatory arthritis; psychosocial disorders limiting rehabilitation; a history of knee arthroplasty (including unicomartmental, biocompartmental, or patellofemoral joint arthroplasty), patellectomy, high tibial osteotomy, or primary TKA in the affected knee; and less than 3 years of postoperative follow-up. Finally, 150 patients (152 knees) were included. The measured resection technique was adopted intraoperatively in 70 patients (70 knees), and the gap-balancing technique assisted by the new balancing device and PSI was adopted intraoperatively in 80 patients (82 knees). We collected patient demographic information (sex, age, BMI), the Knee Society score (KSS), alignment and deformity details preoperatively and the flexion angle of knee, VAS pain score, and radiographic findings at 12 weeks and 36 weeks.

The flexion angle of the knee, Knee Society score (KSS), and VAS pain score were assessed, and radiographs were taken preoperatively and postoperatively at 6 weeks,12 weeks, 1 year, and then annually. The mechanical leg axis hip–knee angle (HKA) was measured on a lower extremity long-standing radiograph. The mechanical axis of the lower limb was measured using digital radiographs and specific software (PACS, BOWEI Electronic Information, Hunan, China). All measurements were performed by an independent physician (Table 1).

**Table 1:** Demographic data and preoperative alignment and deformity statuses

Variable	Total	GB group (*n* = 82)	MR group (*n* = 70)	*p* value
Age	67±11.3	70.2 (55 to 76)	71.1 (52 to 78)	0.42
Sex (female)	152 (62.32%)	62 (75.61%)	48 (68.57%)	
BMI	24.30±3.99	24.12±3.79	24.37±4.01	0.38
KSS	37.13±21.81	39.03±21.92	35.72±19.16	0.31
Preoperative flexion angle	91.9±17.4	92.2°± 15.4°	90.3±17.2°	0.57
Alignment				
Valgus:*n*(mean alignment)	41 (−10.8±6.5)	17 (−7.9±5.1)^a^	24 (−12.7±6.3)^b^	0.34
Neutral:*n*	20	8	12	0.56
Varus:*n*(mean alignment)	91 (6.0±2.1)	57 (5.9±1.7)	34 (6.2±2.3)	0.51

^a^Valgus 10–20°(4 knees) and valgus <10°(13 knees) in GB group

^b^Valgus 10–20°(7 knees) and valgus <10°(17 knees) in MR group

### The new gap-balancing device

**Fig. 1:**
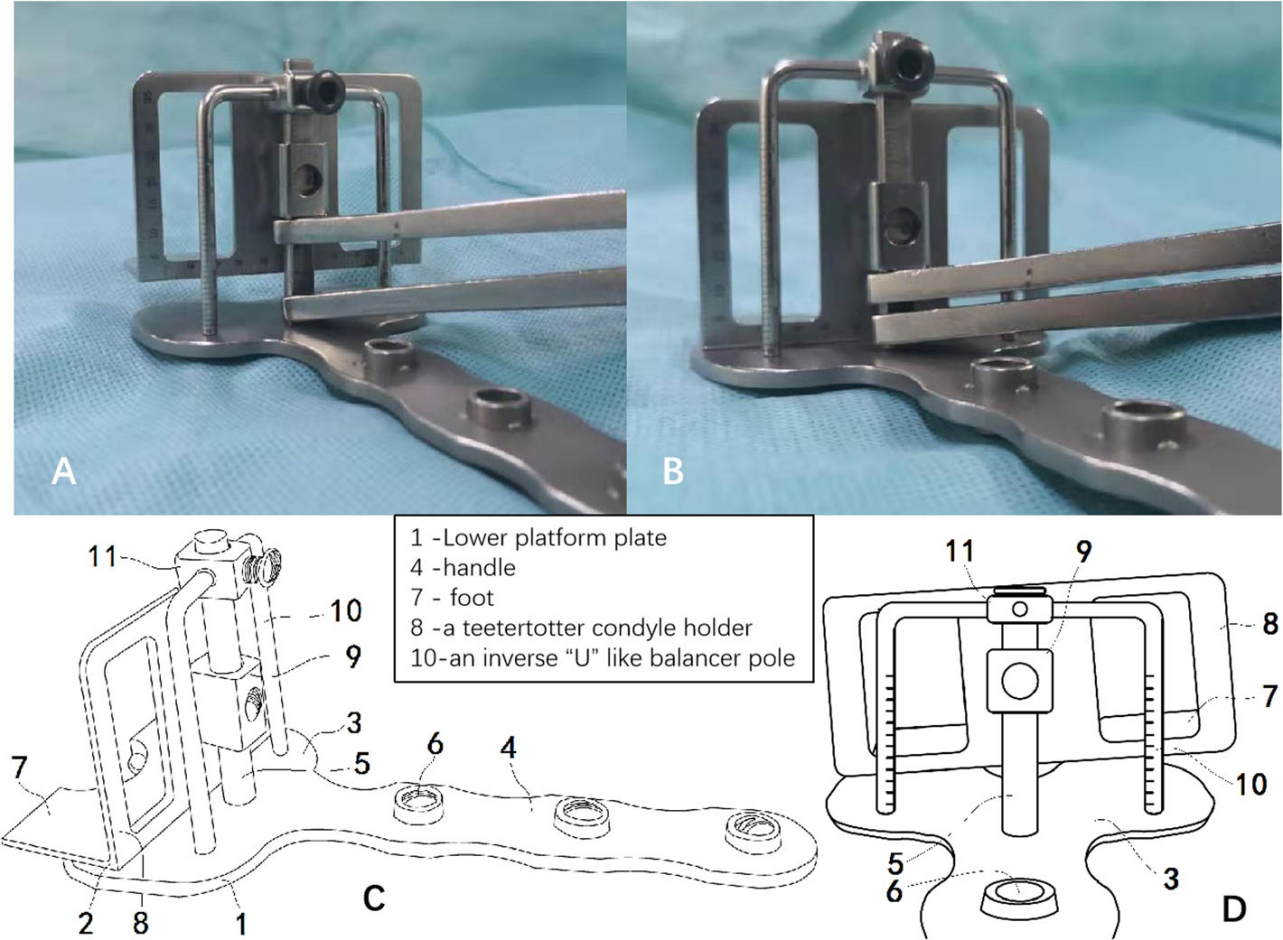
The new gap balancing tool (**a**, **b**) and CAD drawings (**c**, **d**)

The gap-balancing tool was designed by the orthopedic research team of Changsha No. 3 Hospital. The balancing device consisted of three parts: a handle with holes to place the line device connected with a lower platform plate, an inverse “U”-like balancing pole with a scale on both lateral and medial sides to measure the gap and a teetertotter condyle holder. This device was ultimately designed to enable surgeons to find a line—under proper tension in 90° flexion, and the balancing device was sterilized by a plasma sterilizer before being used during the operation. Our group obtained an invention patent or this balancing device (patent number: 201820329898.0) (Fig. 1).

### Preparation of PSI

The patients underwent CT scans before the operation with a 64-row volumetric CT machine (SOMATOM Sensation 40, Siemens, Malvern, PA) and 5-mm slice thickness. The images were stored in DICOM format and analyzed by Mimics 17.0 (Materialise, Belgium). The angle and plate of both tibia and femoral distal bone resection and the prosthetic component size were determined before surgery by the 3D printing technology medical application research institute of Changsha and printed by the Beijing Engineering Technology R&D Center. The objective was to achieve a neutral mechanical axis for the femur and tibia. The plans were reviewed and confirmed by the surgeon for each patient. The resection plates of the tibia were designed to be at a 90°angle to the longitudinal tibial axis with a 3° posterior slope. For ensuring the proximal tibia and distal femur resection can be performed precisely, the contact area between the bone and PSI was enlarged and used the osteophytes as the contact surface as much as possible. The flexion degree in the sagittal plane for the femoral component depended on the patient’s specific anatomical features. The templates were sterilized by a plasma sterilizer before surgery. Our group obtained invention patents for the PSI and design method (patent numbers ZL201520623218.2 and ZL201510507788.X).

### Surgery

A standard midline incision and medial parapatellar arthrotomy were performed using both cruciate-retaining prostheses and posterior stabilized prostheses (Smith & Nephew Legion, LINK GeminiII, and AKMEDICAL A3) in all patients.

#### GB group

**Fig. 2:**
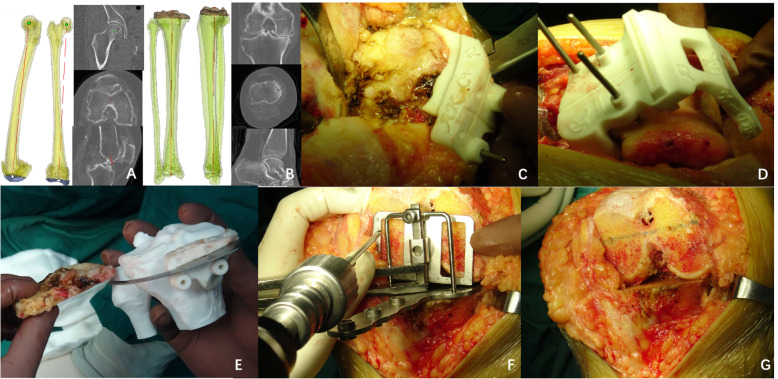
Design of the PSI (**a**, **b**). Resection of the proximal tibia assisted by PSI (**c**); Resection of the distal femur assisted by PSI (**d**). Comparing the thickness of the bone we cut with preoperative planning (**e**). Flexion gap balancing procedure assisted by the new balancing device that we designed (**f**, **g**)

**Fig. 3:**
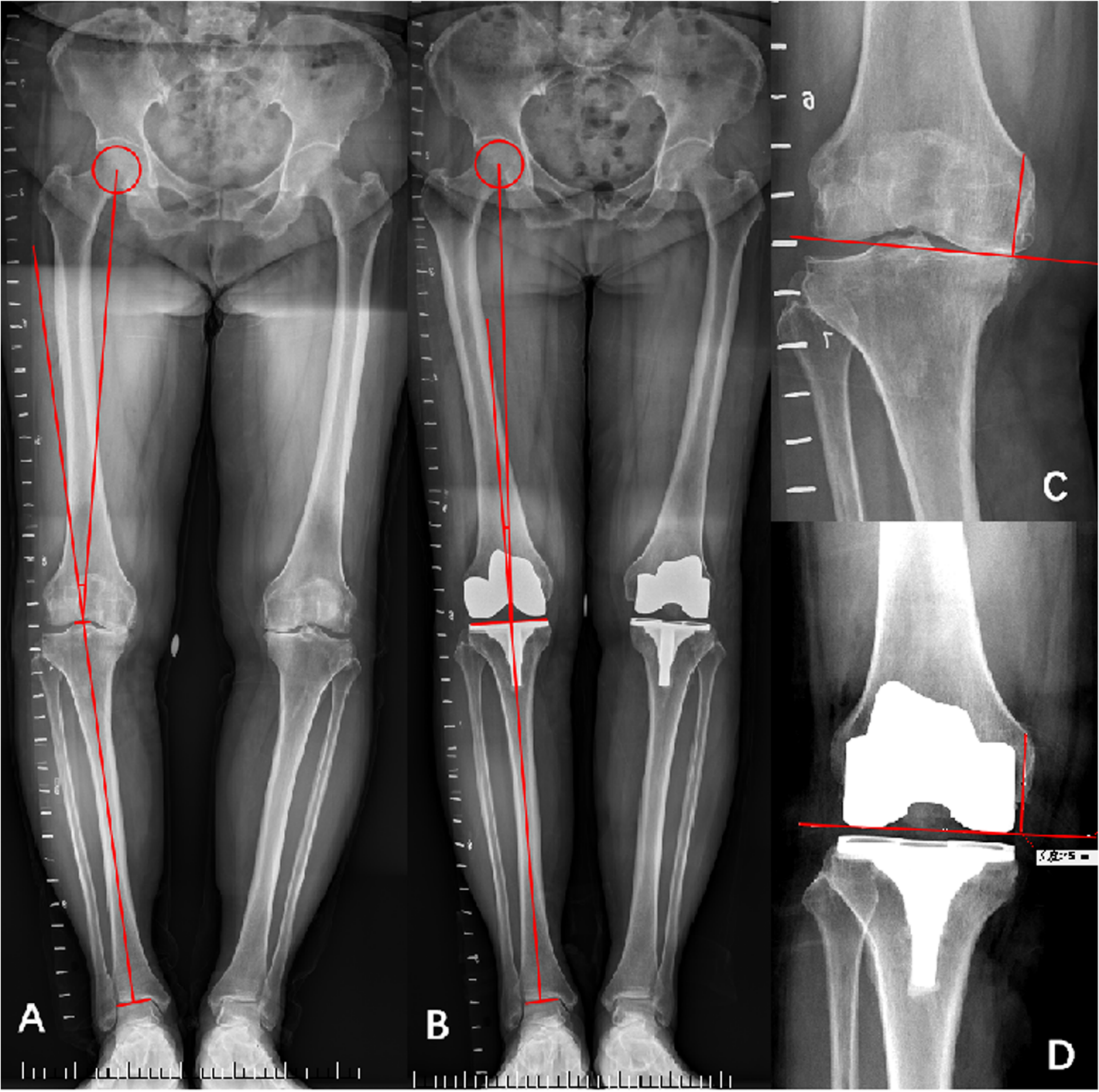
The lower extremity long-standing radiograph and positive X-ray with preoperative and postoperative alignment (red line). HKA is defined as the angle between a line from the femoral head center to the tibial spine center and a line from the tibial spine center to the talus joint surface centre (**a**, **b**). The method of measuring the change of joint line before and after operation with the insertion point of the adductor femur as the reference point (**c**, **d**)

PSI was used to perform resection of the proximal tibia and distal femur. Soft tissues should be completely removed so that the templates can be completely attached to the bone surface as preoperative planning. Only the PSI and bone surface were stable contact without sloshing can the proximal tibia and distal femur resection performed precisely. The tibia was anteriorly dislocated, and the line device was placed to ensure that the osteotomy plane of the tibia was perpendicular to the anatomical axis of the tibia. It is critical to remove all osteophytes before releasing the soft tissue, including the posterior femoral and tibial osteophytes. Part of the posterior condyle was removed if it was difficult to expose the posterior femoral osteophytes. Then, the soft tissue was released to achieve a symmetrical extension gap. No soft tissue was released after this step. The main body and lower platform plate of the balancing device were slid over the proximal tibial cut with the knee flexed at 90°, the condyle holder was firmly placed against the distal femoral cut, and the holder’s foot was placed in contact with the posterior condyles. Then, the lower platform plate and holder were distracted with proper force, and the medial and lateral collateral ligaments became even. The inverse “U”-like balancing pole was placed on the lower platform plate. As tension was applied, the femur rotated, and a line parallel to the proximal tibial cut was made according to the scale on the inverse “U”-like balancing pole. The angle between the holder and the line was recorded as the femur rotation angle (Figs. 2 and 3). An appropriately 4-in-1 resection block was placed parallel to the line mark earlier in the operation, and the block was utilized to perform anterior, posterior, and chamfer bone cuts. The lateral and medial gaps were measured after the balancing device was calibrated.

#### MR group

After adequate exposure of the knee, an extramedullary guide was used to perform resection of the proximal tibia and distal femur. The femur was drilled to introduce an internal femoral alignment rod into the intramedullary canal, followed by a distal cutting block with preset parameters. A posterior referencing cutting block was utilized to identify the ideal component size. Then, an appropriately 4-in-1 resection block was utilized to perform anterior, posterior, and chamfer bone cuts. The trials with predetermined sizes and polyethylene were introduced, and the knee was evaluated for the tracking stability in the AP and varus and valgus planes for balance.

The processes for the tibial plateau, patella, and patellar tracking for both the GB and MR groups were consistent with those traditional surgeries.

### Statistical analysis

The data were stored and analyzed using SPSS 24.0 software (SPSS INC., Chicago, IL, USA). The demographic data are presented as the mean±standard deviation (SD). The categorical variables were compared with the chi-square or Fisher’s exact tests. Differences and correlations of *p*<0.05 were considered statistically significant.

## Results

### Patient outcomes

The follow-up period ranged from 35 to 52 months (mean 45 months). The mean ROM did not significantly differ between the measured resection and gap-balancing groups at 12 weeks (100.2°± 11.3° vs. 99.3°±13.2°, *p*=0.527) or 36 weeks (109.4°± 9.4°vs. 110°±12.1°, *p*=0.490). There were no significant differences between the measured resection and gap-balancing groups in terms of the KSS score (82.75±20.98 vs. 81.29±19.67 points, *p*=0.712), the VAS pain score at 12 weeks (1.57±2.91 vs. 2.67±2.29 points, *p*=0.496), the KSS score (92.19±19.11 vs. 88.17±22.45 points, *p*=0.623), or the VAS pain score at 36 weeks (1.37±2.23 vs. 2.10±2.45 points, *p*=0.414).

### Prosthesis survivorship

At this time, the prosthesis survivorship is 99.5% in the GB group and 100% in the MR group. Only one patient, who was included in the gap-balancing group, underwent a revision surgery at 2 years postoperatively due to infection. At the final follow-up, the patient got a KSS score of 82 points and had no further sequela.

### The accuracy of the PSI and balancing device

The average operation time was 51 min (41–69 min) in the GB group and 58 min (43–67 min) in the MR group, and the difference between groups was not significant (*P*>0.05). The average time required to balance the flexion gap was 2 min. We consider the measuring procedure easy to perform with the help of the gap balancing device we designed. In the GB group, all patients underwent TKA and prosthesis implantation with the PSI, and the balancing device was used for gap balancing as planned. In 3 cases, the tibial component used intraoperatively was one size smaller than planned.

### Complications

After the operation, 4 cases of complications occurred in the GB group (4/82), and 6 occurred in the MR group (6/70); knee anterior pain (2 knees) caused by patella arthritis, joint conglutination (1 knee) and infection (1 knee) occurred in the GB group, and knee anterior pain (5 knees) caused by abnormal patellar tracking and joint conglutination (1 knee) occurred in the MR group. There were no intraoperative complications (Table 2).

**Table 2:** Clinical and radiographic outcome data at 12 weeks and 36 weeks

Variable	GB group (*n* = 82)	MR group (*n* = 70)	*p* value
Joint line displacement (mm)	1.3±1.1	1.2±1.4	0.391
Flexion angle at 12 weeks	100.2°± 11.3°	99.3°±13.2°	0.527
Flexion angle at 36 weeks	109.4°± 9.4°	110°±12.1°	0.490
HKA (°)	1.8°±1.5°	1.4°±1.2°	0.556
Correction varus/valgus angle (°)	7.4°±5.7°	9.3°±7.6°	0.336
KSS at 12 weeks	82.75±20.98	81.29±19.67	0.712
KSS at 36 weeks	92.19±19.11	88.17±22.45	0.623
VAS at 12 weeks	1.57±2.91	2.67±2.29	0.496
VAS at 36 weeks	1.37±2.23	2.10±2.45	0.414

*HKA* hip–knee angle

### Radiographic analysis

The radiographic evaluation at the latest follow-up did not demonstrate any evidence of progressive radiolucencies, loosening, or subsidence of any prosthesis except for in the case of infection. The average joint line displacement was 1.3 ± 1.1 mm (range 0–3) proximally in the GB group and 1.2±1.4 mm in the MR group. No outliers >5 mm in either group were recorded.

The mean leg axis deviation from the neutral mechanical axis was 1.8°±1.5° varus (range 0°–3° varus) in the GB group and 1.4°±1.2° (range 0°–3° varus) in the MR group. No outliers with >3° deviation in either group were recorded (Table 2). The method of measuring joint line displacement and HKA was shown in Fig. 3.

## Discussion

Currently, either the measured resection or gap-balancing technique is used in standard TKA to determine the angle of femoral component rotation. Bone cuts are made to relive soft tissue tension in the measured resection technique. The gap-balancing technique relies on ligament release prior to bone cutting. However, the best method for obtaining rotational alignment of the femoral component during flexion remains controversial. The gap-balancing technique is supported by many surgeons, as it can yield a symmetric, rectangular flexion space intraoperatively. Although the gap-balancing technique is effective, there still exist some limitations, which need be discussed further.

First, the gap-balancing technique has been thought to sacrifice joint-line alignment for gap symmetry. The joint line may be elevated due to significantly greater distal femoral resection and a larger tibial insert thickness [27]. In the measured resection technique, femur and tibial resection are performed independently, whereas in the gap-balancing technique, osteotomy of the femoral anteroposterior condyle is performed with respect to the outcome of proximal tibia resection. Improper tibial resection can also lead to elevation of the joint line or a mismatch of the flexion and extension gap dimensions. Therefore, accurate proximal tibial and femur distal cuts are crucial.

The PSI we adopted in this study has a design feature which was expected to reduce the osteotomy error. The contact area between the bone and PSI was enlarged and used the osteophytes as the contact surface as much as possible. Soft tissues should be completely removed so that the templates can be completely attached to the bone surface as preoperative planning. Only the PSI and bone surface were stable contact without sloshing can the proximal tibia and distal femur resection performed precisely. While there are some disadvantages need to be pointed out, including considerable costs for preoperative scans and production of cutting guides, delay in surgery associated with preoperative CT and radiation exposure associated with CT prototyping. This conclusion is consistent with previous research on PSI [8].

In this study, data from 152 patients who underwent TKA from August 2014 to June 2016 were analyzed retrospectively, and we provide compared the intraoperative parameters of the GB (assisted by PSI and the new balancing device) and MR techniques regarding femoral component placement. Resection of the proximal tibia and distal femur was performed with PSI components, which were designed according to the individual’s anatomical characteristics; the use of PSI has been proven to be safe and lead to good accuracy in some orthopedic surgeries [20, 28–31]. Our research team has extensive experience with digital orthopedics [32]. In this study, the tibial components did not have deviations >1.5° from the preoperative plans. There were no relevant cases of displacement of the joint line >3 mm in either group; there were 15 cases of displacement of 3 mm in the GB group and 12 cases in the MR group, and the magnitude of the displacement was ≤2 mm in the remaining knees. Additionally, the tibial component deviated by <1.5° from the plans when PSI was used.

Another critical aspect of the gap-balancing technique is femoral component rotation. Many principles and surgical devices for ligament balance during TKA have been developed. Different spacers, including trial components and blocks, may assist in stretching the ligaments. The medial and lateral lift-off can then be measured visually by the surgeon based on his or her experience or indirectly by a navigator. Tensors and spreaders apply tension to the ligaments in a controlled manner with or without electric instruments to measure compressive loads. Most of these devices are expensive, increase the complexity of the surgery, and are time-consuming.

The new balancing device we designed and used in surgery is like a seesaw; it was used to find the bony landmark parallel to the posterior condylar re-resection plate and confirm femoral component rotation. We consider that the greatest advantage of the balancing device is that it makes it easy to perform the flexion balance procedure, and it takes no more than 2–3 min to confirm femoral component rotation. The scale on the condylar holder clearly shows the lateral and medial gap heights. The clinical and radiographic outcome data recorded at 12 weeks and 36 weeks demonstrated that patients can exhibit satisfactory function after undergoing surgery with the gap-balancing technique assisted by PSI and the new balancing device. There are some disadvantages that need to be addressed and overcome: we cannot determine the joint distraction force explicitly, and the use of a grip dynamometer connected to the balancing device may improve precision.

In this study, the knee with maximum valgus angle is 17°, only 4 knees with valgus knee ranged between 10° and 20° in the GB group and 7 knees in the MR group. The rest of the knee varus/valgus angle was less than 10°. All the varus and valgus knee deformities can be corrected to neutral alignment by intraarticular osteotomy and soft tissue release techniques. So it is not clear if the gap-balancing device can be applied in the case of severe varus or valgus deformity. Consequently, more scientific and valuable research needs to be performed.

We achieved the goals of avoiding anatomical differences, finding an easy way to obtain a symmetric, rectangular flexion space intraoperatively and restoring the natural joint line by using PSI and the balancing device in this study. Our analysis showed that both the measured resection and combination techniques can be used to achieve accurate femoral component alignment and similar 3-year outcomes. The results are consistent with those of previous studies [33, 34].

There are some limitations of this study. First, it is a retrospective study, and we cannot compare the femoral component rotation angle with the bony markers. Second, the follow-up period was relatively short. Consequently, more scientific and valuable research needs to be performed.

## Conclusion

In conclusion, the gap-balancing technique assisted by the new balancing device and PSI can yield accurate femoral component alignment as well as outcomes similar to those of measured resection at 3 years. The new balancing device can be considered by surgeons who prefer the gap-balancing technique together with the PSI.

## Data Availability

Not applicable.
